# Self-Weighted Convolutional Neural Networks for RF Signal Classification in Support of Wireless Spectrum Awareness

**DOI:** 10.3390/s26196186

**Published:** 2026-09-29

**Authors:** Xihui Zhang, Xinyuan Zhang, Wenke Li, Jianqing Li, Jiaxu Liu

**Affiliations:** 1 Southwest China Institute of Electronic Technology, Chengdu 610036, China; 2 Department of Electronic Science and Engineering, University of Electronic Science and Technology of China, Chengdu 611731, China; 3 The Fifth Research Institute of Telecommunications Technology, Chengdu 610062, China

**Keywords:** automatic modulation recognition, convolutional neural network, channel scaling, deep learning, transfer learning

## Abstract

RF signal classification provides modulation information that can support wireless spectrum awareness after signal acquisition and segmentation. This paper presents a self-weighted convolutional neural network for classifying communication-signal modulation formats from in-phase/quadrature (IQ) sequences. In the proposed multi-branch block, the normalized output channels of one convolutional branch are multiplied by learnable scaling coefficients before aggregation. Stacked blocks are followed by squeeze-and-excitation recalibration and a fully connected classification head. The network is evaluated on a MATLAB-generated 12-class IQ dataset under variations in network depth, symbol rate, carrier-frequency offset, and transfer settings, and is compared with Inception, ResNet50, CaiT, and ViT for Small-Size Datasets. An institute-provided IQ dataset with the same modulation categories provides an additional within-dataset evaluation. On the synthetic dataset, the proposed network achieves an average test accuracy of 91.91%, 0.52 percentage points above Inception. Fine-tuning on a small target-domain subset achieves 91.81% average accuracy while reducing the observed convergence requirement from 21 to 5 epochs compared with training from scratch on the target dataset. On the institute-provided dataset, the proposed, transfer, and Inception networks achieve 93.61%, 92.75%, and 92.32%, respectively. These results support the proposed method as a modulation-aware RF signal-classification component for wireless spectrum-awareness workflows under the evaluated synthetic and institute-provided data settings.

## 1. Introduction

Automatic modulation recognition (AMR) identifies the modulation format of a received signal without complete transmitter-side information. It is important in spectrum monitoring, interference analysis, cognitive radio, and non-cooperative communication systems, where reliable modulation information can support situational awareness and signal management. Classical AMR methods are generally likelihood-based or feature-based. Likelihood-based approaches can approximate optimal Bayesian decisions when accurate signal and channel models are available, but their computational cost and sensitivity to nuisance-parameter estimation can be substantial. Feature-based approaches reduce this burden by extracting expert-designed statistics, spectra, or cyclostationary features, at the cost of task-specific feature engineering [1,2,3,4,5].

Deep learning replaces part of this handcrafted pipeline with trainable representations [6]. AMR studies have adopted several input forms, including constellation images and related feature maps, raw in-phase/quadrature (IQ) sequences, amplitude-phase sequences, frequency-domain sequences, and fused multi-representation inputs [7,8,9,10,11,12,13,14,15,16]. The input representation determines which signal structures are directly available to the model and influences the required preprocessing, sequence length, computational cost, and robustness to impairments. It also affects the extent to which prior signal-processing knowledge is embedded in the input rather than learned from data. Accordingly, convolutional, recurrent, residual, and densely connected networks have all been investigated in modulation-classification settings [17,18,19,20,21,22,23,24].

Image-based AMR converts received samples or derived statistics into two-dimensional representations, such as constellation diagrams, time–frequency maps, and higher-order feature images. Convolutional networks can then exploit spatial patterns in these representations. For example, constellation-image classifiers and dimensionality-reduction approaches have been studied to learn discriminative visual features while controlling the computational burden [7,17,18]. Generative methods have also been used to enrich constellation-based representations [8]. These approaches can make certain modulation-dependent structures visually explicit; however, the transformation from a waveform to an image may discard temporal information or introduce representation-specific design choices.

Raw IQ sequences preserve the temporal evolution of the received baseband waveform and are therefore widely used for end-to-end AMR. Early work demonstrated deep-network recognition directly from radio signals and established commonly used benchmark-style IQ datasets [9,10]. Subsequent studies evaluated convolutional, residual, densely connected, recurrent, and convolutional-recurrent architectures for extracting local waveform patterns and longer-range temporal dependencies [19,20,22,23,24]. For these models, the receptive field, sequence length, and channel capacity jointly determine the balance between preserving signal detail and controlling computational complexity. CNN-based models offer parallel computation and effective local feature extraction, whereas recurrent components can model sequential dependence but may increase training and inference cost. Hybrid CNN–LSTM and related architectures seek to balance these characteristics [22,23,24]. Recent work has further investigated causal convolution with structural reparameterization for compact, noise-robust recognition and contrastive learning for transferability at low signal-to-noise ratios and robustness to uncertain noise [25,26].

Channel and receiver impairments remain a major challenge because fading, noise, carrier-frequency offset, and phase offset can alter the observed waveform and reduce class separability. Learnable preprocessing and distortion-correction modules have been used to compensate for frequency and phase offsets [27,28,29,30]. Other studies have considered long-sequence processing, feature fusion, uncertain noise, and cooperative reception to improve robustness under varied signal conditions [13,31,32,33]. These factors make a model trained under one signal setting potentially sensitive to changes in receiver configuration or channel distribution. In addition to IQ inputs, amplitude-phase and frequency-domain sequences provide alternative descriptions of the received signal and can be useful when their corresponding structures are informative for the target modulation classes [11,12].

Feature fusion and transfer learning provide complementary routes for dealing with heterogeneous or changing signal conditions. Multi-representation methods combine IQ sequences with derived features or images to exploit information unavailable in a single representation [13,14,15,16]. Transfer learning and domain adaptation can adapt a classifier when signal or channel distributions change and only limited target-domain data are available [34,35]. Recent domain-adaptation work has also considered superimposed domain differences, in which multiple mismatch factors occur together [36]. Such adaptation is particularly relevant when channel conditions, receiver hardware, or signal parameters differ between a source setting and the target setting. Related radio-frequency identification studies have used multi-domain migration, iterative domain adaptation, and adversarial continual learning to address temporal and domain shifts [37,38,39]. These results motivate a careful examination of parameter transfer in AMR, but they do not by themselves establish performance for the present task.

Multi-branch convolutional networks such as Inception process an input through parallel transformations before aggregation [40], while residual connections facilitate optimization by preserving direct information and gradient paths [41]. This motivates architectures that preserve complementary local features before deciding how to aggregate them. Building on these ideas, the network studied here applies a learnable coefficient to each output channel of one convolutional branch before branch aggregation. The subsequent squeeze-and-excitation (SE) block performs input-dependent channel recalibration after aggregation [42]. The contributions of this paper are as follows:

1. A stacked one-dimensional convolutional architecture is formulated in which the normalized output of one branch is multiplied by a learnable channel-wise scaling vector before aggregation.
2. Exploratory experiments report the sensitivity of the resulting classifier to feature-head depth, backbone depth, symbol-rate variation, and carrier-frequency offset on a synthetic 12-class IQ dataset.
3. A parameter-transfer experiment compares fine-tuning on a small target-domain subset with training from scratch on the target dataset and reports comparable accuracy with fewer convergence epochs.
4. The experiments compare the proposed and transfer networks with Inception, ResNet50, CaiT, and ViT for Small-Size Datasets; the corresponding accuracy curves and summary statistics are provided for the synthetic setting.

The remainder of the paper describes the signal and dataset model, the proposed architecture, the experimental setup, the results, and the study limitations.

## 2. Materials and Methods

### 2.1. Signal and Dataset Model

The study uses synthetic complex-baseband signals generated in MATLAB. For modulation class *m*, the received signal can be represented generically as

(1) $$r_{m}\left(t\right)=\left[h\left(t\right)*s_{m}\left(t\right)\right]e^{j2\pi \Delta ft}+n\left(t\right),$$

where $s_{m}\left(t\right)$ denotes the transmitted baseband waveform, h(t) is the multipath channel impulse response, Δf is the carrier-frequency offset, n(t) is additive noise, and * denotes convolution.

The dataset contains BPSK, QPSK, 8PSK, OQPSK, π/4-QPSK, MSK, GMSK, 8QAM, 16QAM, 32QAM, 2FSK, and 4FSK. Unless otherwise specified, each waveform was generated with unit average symbol power, four samples per symbol, root-raised-cosine pulse shaping for the PSK and QAM classes, and additive white Gaussian noise at the specified SNR. For each class, 21,000 samples cover the SNR range from 0 to 20 dB in 1 dB increments, with 1000 samples per SNR value. The full dataset therefore contains 252,000 samples, which are divided into training, validation, and test subsets in an 80%/10%/10% ratio as shown in Table 1, Table 2 and Table 3.

### 2.2. Institute-Provided IQ Dataset

An IQ dataset provided by a research institute was used for an additional evaluation. The institute name and waveform records are withheld for privacy reasons. The dataset uses the same 12 modulation categories as the synthetic dataset and is represented as two-channel in-phase/quadrature sequences. For each modulation class, 3200 records were divided using a fixed stratified 8/1/1 split, corresponding to 2560, 320, and 320 records for training, validation, and testing, respectively. All methods used this within-dataset split and the same preprocessing (see Table 4).

### 2.3. Proposed Network

**Figure 1:**
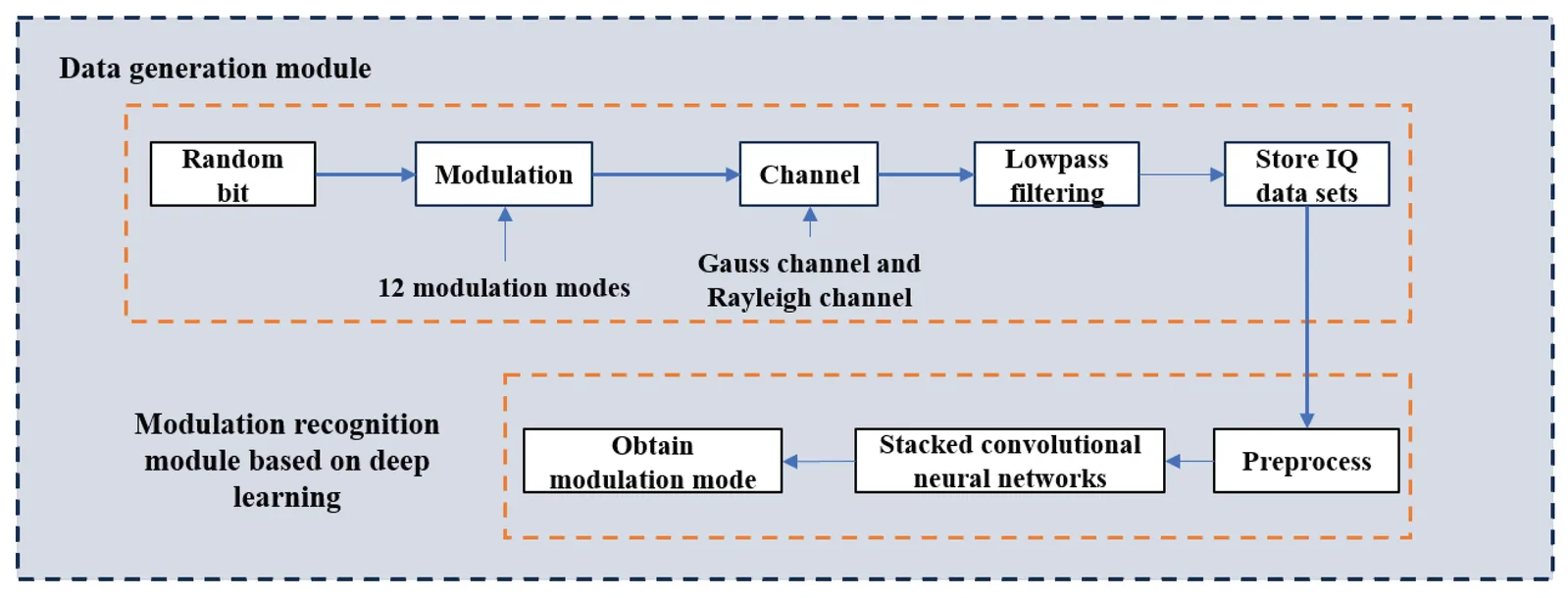
Workflow for synthetic IQ generation, preprocessing, network training, and modulation prediction.

The processing workflow is shown in Figure 1. Random source symbols are modulated, passed through the synthetic channel model, low-pass filtered, and stored as IQ examples. Each example is associated with its modulation label. After normalization, the IQ examples and labels are used to train the classifier, and the trained network produces modulation predictions for the corresponding test examples. This workflow links the signal-generation settings, representation learning, and final recognition evaluation without assuming that the synthetic channel reproduces all operational environments.

At the architectural level, the classifier consists of five connected stages: a two-channel IQ input, a repeated self-weighted convolutional backbone, channel processing through learnable scaling and SE recalibration, a global average pooling (GAP)-based feature head, and a softmax decision stage. The following description specifies how these stages are connected while retaining the same data flow for all experiments.

### 2.4. Self-Weighted Convolutional Block

**Figure 2:**
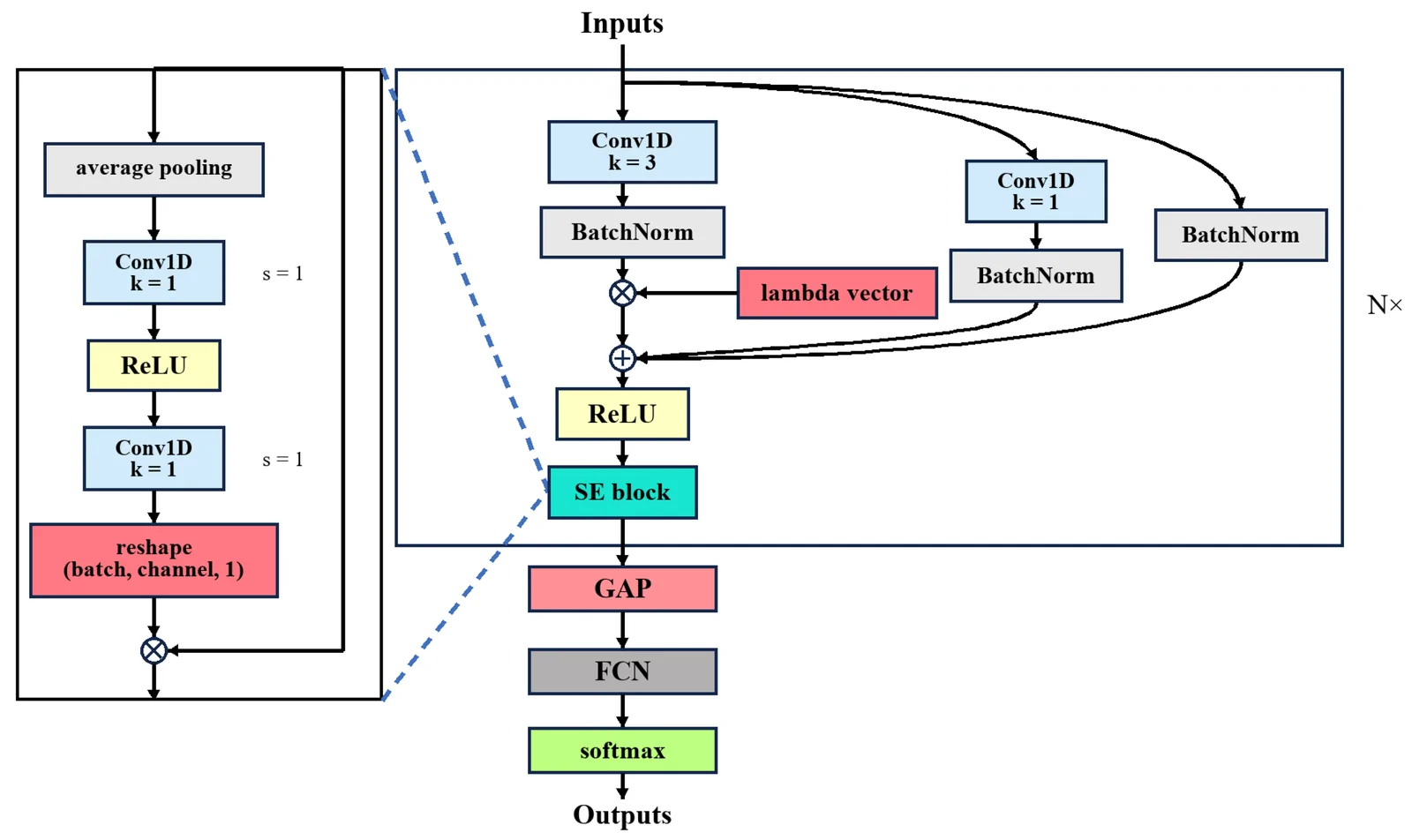
Architecture of the self-weighted one-dimensional convolutional network. The learnable scaling vector is applied only to the normalized output of the one-dimensional kernel-size-3 branch before aggregation.

Figure 2 shows the proposed architecture. The input is a two-channel one-dimensional array with shape (B,2,L), where *B* is the batch size and *L* is the sequence length. The backbone stacks *N* self-weighted blocks, followed by GAP, fully connected layers, and a softmax decision at inference time. The implementation settings used for the final classifier are summarized in Table 5.

For block *l*, let $C_{l}$ be the number of output channels in the first branch. The trainable scaling vector is

(2) $$\lambda_{l}={\left[\lambda_{l,1},\ldots ,\lambda_{l,C_{l}}\right]}^{T}.$$

The three branch outputs are written as

(3) $$\begin{array}{cc} u_{l}^{\left(1\right)} & ={\mathrm{BN}}_{l}^{\left(1\right)}\left({\text{Conv1D}}_{k=3,l}\left(x_{l}\right)\right), \end{array}$$

(4) $$\begin{array}{cc} u_{l}^{\left(2\right)} & ={\mathrm{BN}}_{l}^{\left(2\right)}\left({\text{Conv1D}}_{k=1,l}\left(x_{l}\right)\right), \end{array}$$

(5) $$\begin{array}{cc} u_{l}^{\left(3\right)} & ={\mathrm{BN}}_{l}^{\left(3\right)}\left(P_{l}(x_{l}\right)). \end{array}$$

where $P_{l}$ is the identity mapping when the input and output channels match and a 1×1 projection otherwise. Aggregation is

(6) $$z_{l}=\mathrm{ReLU}\left(\lambda_{l}⊙u_{l}^{\left(1\right)}+u_{l}^{\left(2\right)}+u_{l}^{\left(3\right)}\right),$$

where ⊙ denotes channel-wise multiplication with broadcasting over the temporal dimension. In the implementation, the batch-normalization layers in the three branches use running statistics but do not use trainable affine parameters; consequently, the learnable vector $\lambda_{l}$ is not absorbed by a branch-wise BN scale parameter. The vector is initialized as $\lambda_{l}=1$ so that the initial block behaves like an unweighted multi-branch aggregation, and it is placed in a separate optimizer parameter group with zero weight decay. The SE block then produces $x_{l+1}={\mathrm{SE}}_{l}\left(z_{l}\right)$. The formulation distinguishes the static trainable scaling vector from the input-dependent SE response.

### 2.5. Feature Extraction and Classification

After the stacked backbone, GAP aggregates the temporally indexed feature maps into a fixed-length representation. This separates temporal feature extraction in the backbone from the mapping of global features to modulation classes in the classifier head. The architecture uses three fully connected layers, ReLU after the first two layers, and dropout with a rate of 0.15. The final layer produces 12 logits. During training, the logits are passed directly to the cross-entropy objective. The softmax function is used only to convert logits to class probabilities for inference.

### 2.6. Experimental Setup

The experiments use min–max normalization,

(7) $$Y_{i}=\frac{X_{i}-X_{\min}}{X_{\max}-X_{\min}},$$

and all IQ inputs were normalized before model training and evaluation. The normalization was applied independently to the in-phase and quadrature channels of each IQ example, with $X_{\min}$ and $X_{\max}$ computed from the corresponding channel within that example. The same normalization rule was used for the training, validation, and test subsets, and no statistics from the test subset were used for model selection.

The networks were trained with categorical cross-entropy,

(8) $$L=-\frac{1}{N_{b}}\sum_{n=1}^{N_{b}}\sum_{m=1}^{M}y_{n,m}\log\left(\frac{\exp(q_{n,m})}{\sum_{i=1}^{M}\exp\left(q_{n,i}\right)}\right),$$

where $N_{b}$ is the batch size, M=12 is the number of classes, $q_{n,m}$ is the logit for class *m*, and $y_{n,m}$ is the one-hot target. Training used Adam optimization with a cosine-annealing learning-rate schedule. Adam was selected because its adaptive first- and second-moment estimates provide stable optimization for the convolutional and channel-scaling parameters, while the cosine-annealing schedule gradually reduces the learning rate during later training and avoids an abrupt stepwise decay. The training configuration is given in Table 6. Unless otherwise stated, the same optimizer, batch size, maximum epoch count, validation-based checkpoint selection rule, and test protocol were used for the proposed network and all comparison architectures.

Experiments were implemented in PyTorch 2.4.0 on an NVIDIA GeForce RTX 3080 Ti GPU and an Intel Xeon Bronze 3204 CPU under Ubuntu 20.04.2 LTS.

### 2.7. Comparison Architectures

The proposed network and its transfer-learning variant are compared with stacked Inception, ResNet50, CaiT, and ViT for Small-Size Datasets. The comparison spans three representative topology families: multi-branch convolution, residual convolution, and Transformer-based recognition. Inception provides a multi-branch convolutional reference [40], and ResNet50 provides a residual convolutional reference [41]. ViT introduces patch-based self-attention [43]; CaiT uses class-attention processing and LayerScale [44], while ViT for Small-Size Datasets uses shifted patch tokenization and locality self-attention [45]. This selection places the proposed architecture in a broad structural context rather than treating any single baseline as definitive. The same dataset partition and evaluation criterion were used for all methods within each experiment.

## 3. Results and Discussion

### 3.1. Feature-Head Depth

This sensitivity analysis examines whether the classification head has sufficient capacity to map backbone features to modulation labels, rather than attributing all performance differences to the convolutional backbone. Table 7 compares the number of stacked backbone blocks and fully connected layers. One fully connected layer yields substantially lower accuracy than two or three layers. The difference between two and three layers is small at several backbone depths; for three backbone blocks, the two-layer head is 0.28 percentage points higher.

An isolated feature head was also evaluated after an embedding convolution. Table 8 gives the configuration. Its average accuracy was 23.60%, indicating that the tested head alone did not replace the backbone representation.

### 3.2. Backbone Depth

This analysis examines how the number of repeated self-weighted blocks relates to observed recognition accuracy and validation loss. It therefore provides an exploratory view of the depth-performance trade-off under the fixed experimental setting. Table 9 reports accuracy and average validation loss as the number of blocks increases. Accuracy plateaus near 91.5% for the deeper configurations, while the lowest validation loss occurs at 18 blocks. The 18-block architecture was therefore selected for the subsequent experiments.

### 3.3. Symbol-Rate Variation

**Figure 3:**
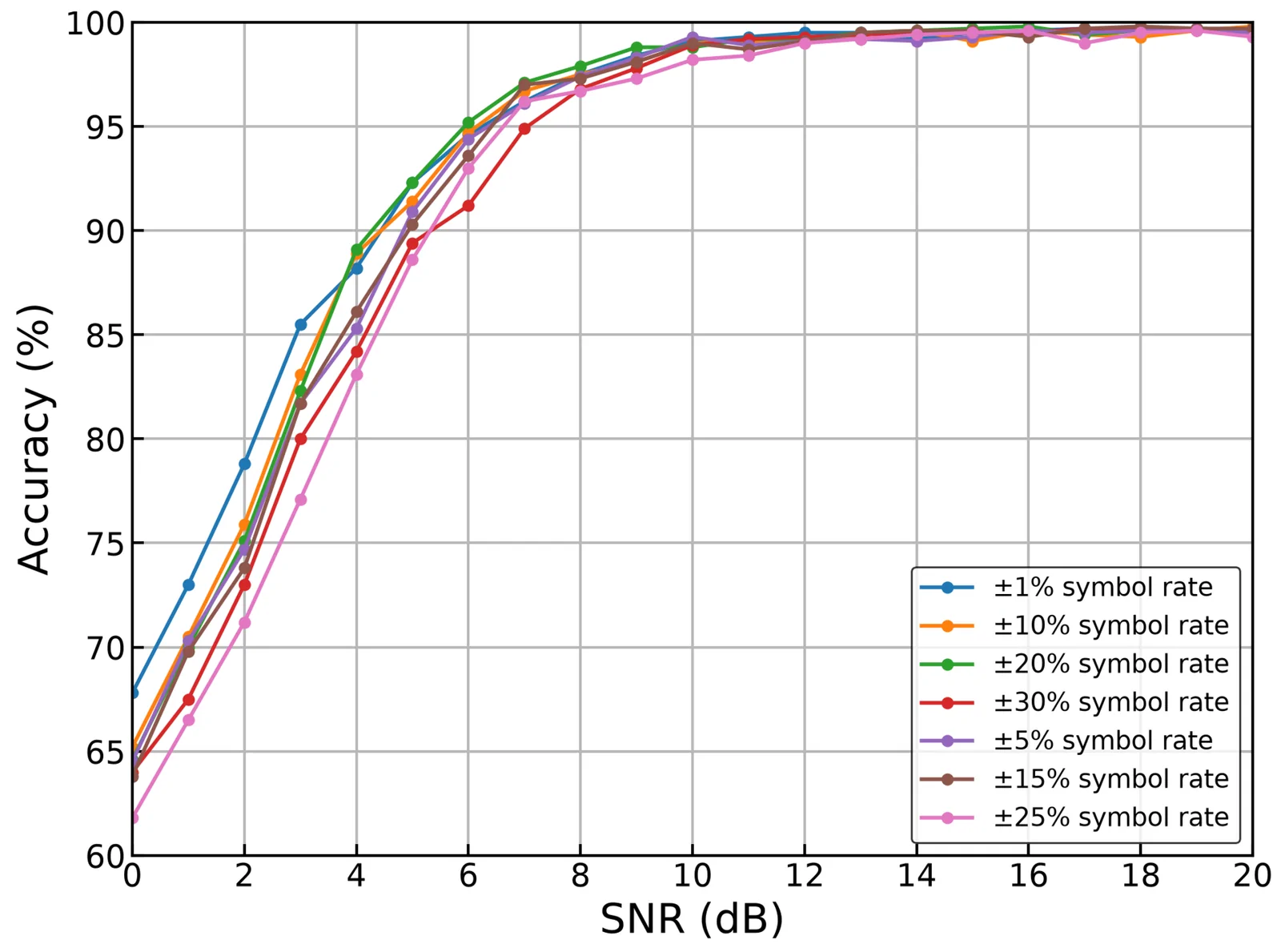
Recognition accuracy versus SNR for seven synthetic symbol-rate ranges centered at 100 kHz. Carrier-frequency offset was set to zero.

Seven synthetic datasets were generated with the same sampling rate and sequence length but different symbol-rate ranges centered at 100 kHz. Carrier-frequency offset was set to zero for this comparison. Figure 3 shows that the curves largely converge above approximately 12 dB, whereas the widest symbol-rate ranges perform less well at lower SNR. The curves for the ±10%, ±15%, and ±20% ranges differ by at most 0.47 percentage points in average accuracy.

### 3.4. Carrier-Frequency Offset

**Figure 4:**
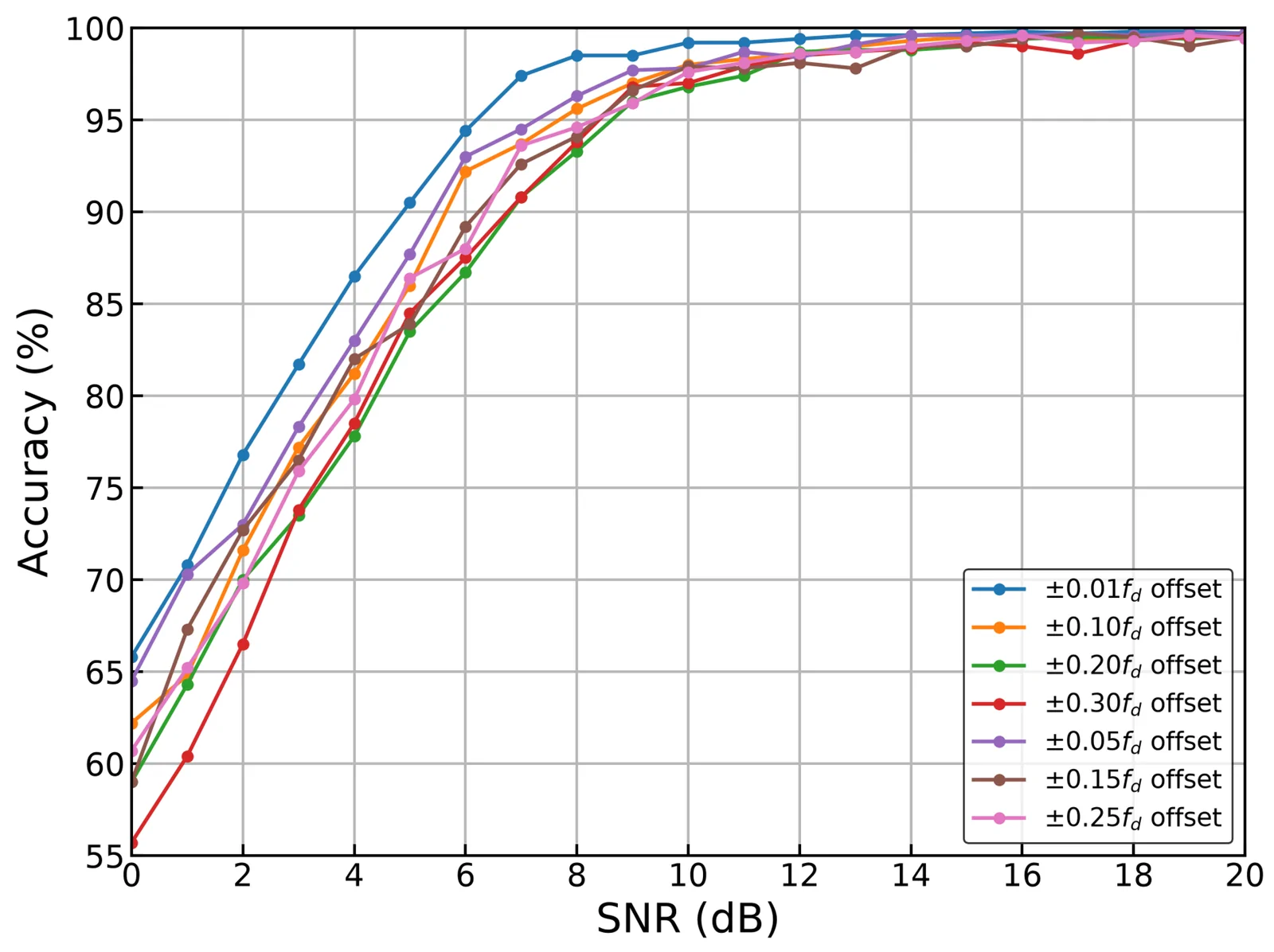
Recognition accuracy versus SNR for carrier-frequency-offset ranges expressed in the legend as normalized fractions of the nominal symbol rate $f_{d}$.

Figure 4 shows accuracy for datasets generated with progressively wider carrier-frequency-offset ranges. Narrower ranges produce higher accuracy, particularly at low and moderate SNR. The average accuracy decreases as the range widens in this experiment.

### 3.5. Transfer Learning Across Signal Settings

**Figure 5:**
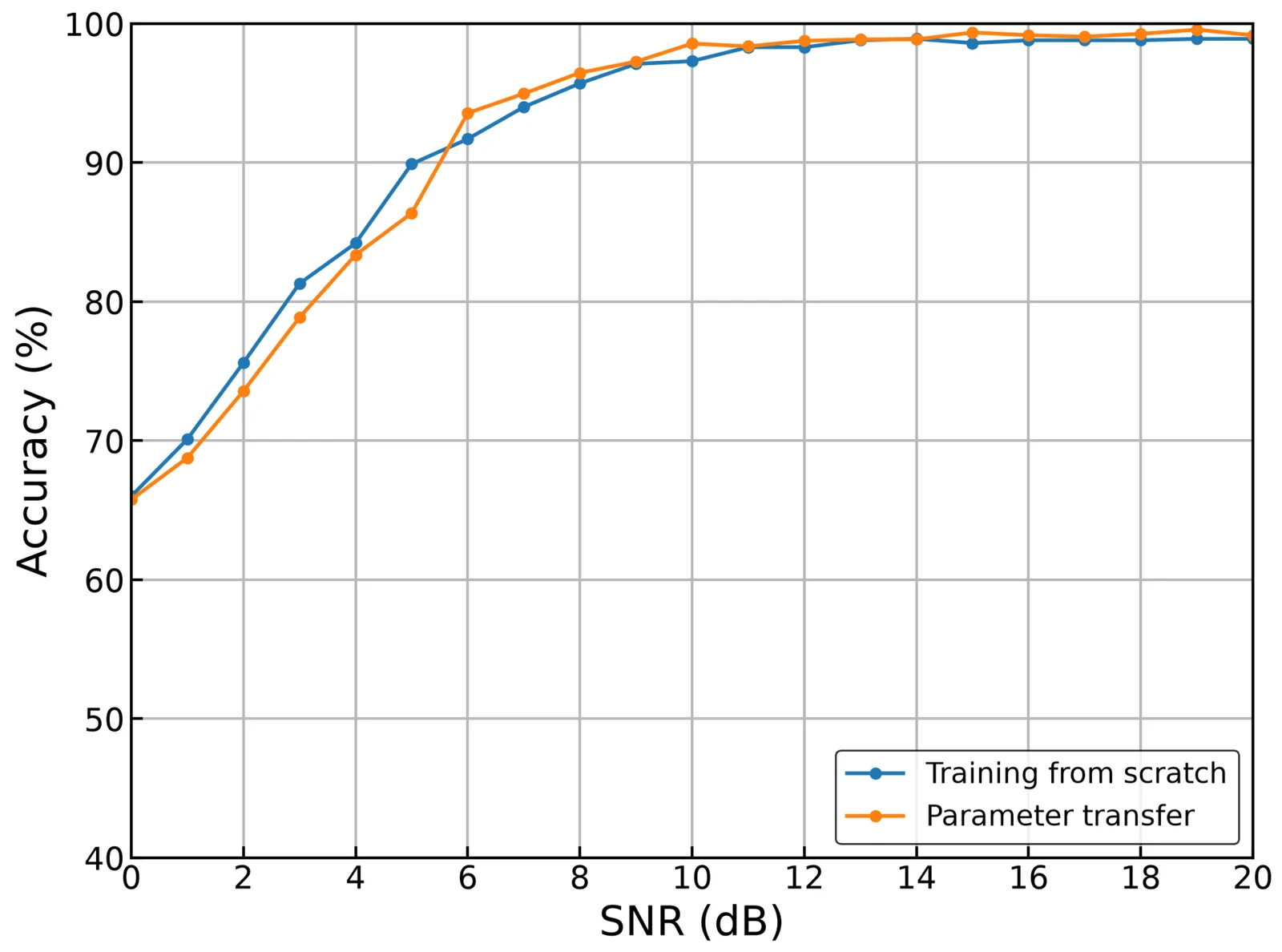
Recognition accuracy versus SNR for training from scratch on the target dataset and fine-tuning on a small target-domain subset.

The transfer experiment used a pretrained model from a broader signal-parameter setting and fine-tuned it using a small subset from the target setting. Figure 5 shows that fine-tuning on the small target-domain subset produced an accuracy curve close to training from scratch on the target dataset across the evaluated SNR range. Table 10 reports the best validation loss, convergence epochs, and test accuracy for the two strategies. Fine-tuning reached 91.81% test accuracy, 0.10 percentage points below training from scratch, while requiring 5 convergence epochs compared with 21 for training from scratch. This corresponds to 23.8% of the epochs, or a 76.2% reduction in epoch count.

### 3.6. Comparison with Existing Architectures

**Figure 6:**
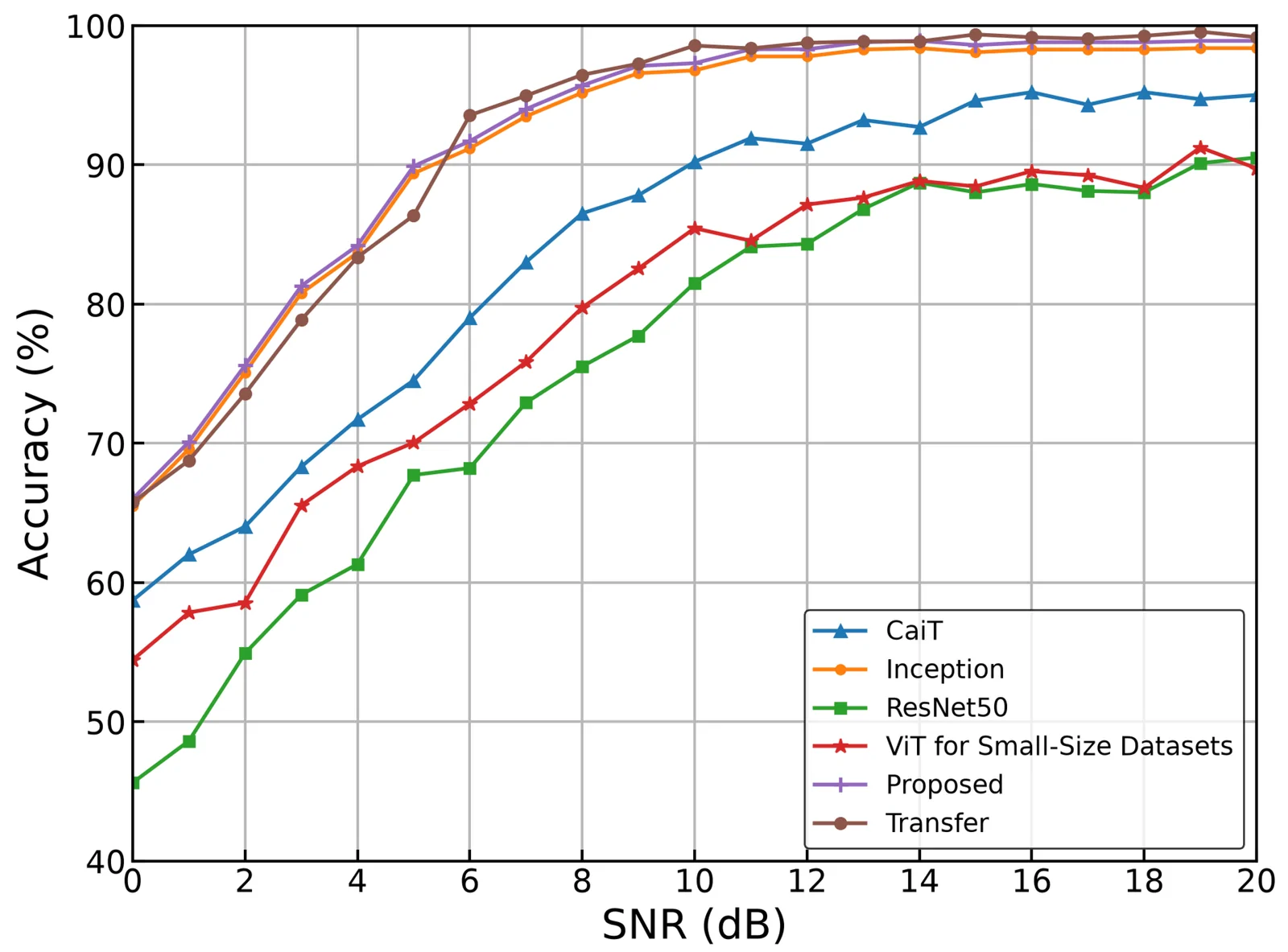
Recognition accuracy versus SNR for the proposed, transfer, and comparison architectures on the synthetic dataset.

Figure 6 and Table 11 compare the proposed and transfer networks with stacked Inception, CaiT, ViT for Small-Size Datasets, and ResNet50. The comparison tests whether the observed performance is retained when the proposed architecture is viewed alongside multi-branch, residual, and Transformer-based alternatives; it does not by itself isolate the contribution of the learnable scaling vector. The proposed network is 0.52 percentage points higher than stacked Inception in average accuracy; the transfer network is 0.42 percentage points higher than Inception and 0.10 percentage points lower than the proposed network trained from scratch.

### 3.7. Evaluation on the Institute-Provided Dataset

The institute-provided dataset provides an additional within-dataset assessment using the same modulation categories as the synthetic data. The comparison includes the proposed network, transfer network, Inception, CaiT, ViT for Small-Size Datasets, and ResNet50, matching the synthetic-data comparison in Table 11. Each method was trained, validated, and tested using the fixed stratified split in Table 4. Table 12 reports aggregate test accuracy.

The proposed network achieves the highest accuracy on the institute-provided dataset, followed by the transfer and Inception networks. Its margin over Inception is 1.29 percentage points, and the ordering of the six methods is consistent with the synthetic-data comparison.

## 4. Conclusions

This paper presents a stacked one-dimensional convolutional network that applies learnable channel-wise scaling to one normalized branch before multi-branch aggregation. The proposed method is evaluated as an RF signal-classification component that can provide modulation information for wireless spectrum-awareness workflows after signal acquisition and segmentation. On the synthetic 12-class dataset, the final model achieves 91.91% average accuracy, compared with 91.39% for stacked Inception. Fine-tuning on a small target-domain subset achieves 91.81% average accuracy and reduces the observed convergence requirement from 21 to 5 epochs compared with training from scratch on the target dataset. On the institute-provided dataset, the proposed network achieves 93.61% accuracy, exceeding the transfer and Inception networks by 0.86 and 1.29 percentage points, respectively; the ranking of all six evaluated methods is consistent with the synthetic-data comparison. The symbol-rate and carrier-frequency-offset studies further show lower recognition accuracy as the corresponding synthetic parameter ranges widen.

The reported results concern modulation classification from IQ signal segments and do not constitute an evaluation of end-to-end signal detection, false-alarm performance, spectrum-occupancy estimation, or wideband spectrum scanning. Within this scope, the results support the use of self-weighted convolutional blocks for modulation-aware RF signal classification under the evaluated conditions. Future work will integrate the classifier with upstream sensing and segmentation stages and will include multi-run evaluation, fixed-weight ablation, and broader validation under additional signal and channel conditions.

## Figures and Tables

**Table 1:** Synthetic dataset parameters.

Parameter	Value
Modulation classes	BPSK, QPSK, 8PSK, OQPSK, π/4-QPSK, MSK,
	GMSK, 8QAM, 16QAM, 32QAM, 2FSK, 4FSK
Symbol rate $f_{d}$	100 kHz
Sampling rate $f_{s}$	400 kHz
Sampling points per example	10,000
Raised-cosine roll-off factor α	0.3
Carrier-frequency offset	$\left[-0.01\;f_{d},\;0.01\;f_{d}\right]$
SNR	0–20 dB, 1 dB increments
Samples	21,000 per class; 252,000 total
Train/validation/test split	80%/10%/10%

**Table 2:** Additional synthetic waveform-generation details.

Item	Setting
Pulse shaping for PSK/QAM	Root-raised-cosine filtering, roll-off factor 0.3, span 10 symbols
Samples per symbol	4
Symbol mapping	Gray coding for PSK/QAM classes where applicable
FSK tone spacing	Orthogonal spacing, $\Delta \;f_{\mathrm{FSK}}=0.5\;f_{d}$ for 2FSK and 4FSK
MSK/GMSK modulation index	h=0.5
GMSK Gaussian filter	Bandwidth-time product BT=0.3, span 4 symbols
Symbol-rate variation study	Symbol rate fluctuates within a range of up to 25%; carrier-frequency offset fixed to zero
Carrier-frequency-offset study	Carrier-frequency offset fluctuates within a range of up to 25%; symbol rate fixed at 100 kHz
Train/validation/test distribution	Same SNR grid, modulation classes, and parameter ranges used in all three splits

**Table 3:** Channel parameters.

Channel	Parameter	Value
AWGN	SNR	0–20 dB
Rayleigh	Number of paths	6
Rayleigh	Multipath delay	0–10 μs
Rayleigh	Multipath gain	−15–0 dB
Rayleigh	Maximum Doppler shift	10 Hz

**Table 4:** Characteristics of the institute-provided IQ dataset.

Parameter	Value
Data provider	Anonymous research institute (privacy protected)
Data representation	Two-channel IQ sequence
Modulation classes	Same 12 classes as the synthetic dataset
Samples per class	3200
Sequence length	8192 IQ samples
Sampling rate	2 MHz
SNR range	5–15 dB
Evaluation protocol and split	Within-dataset stratified split: 2560/320/320 samples per class

**Table 5:** Implementation details of the self-weighted network.

Component	Setting	Notes
Input	Two-channel IQ sequence with shape (B,2,L)	L=10,000 for the synthetic dataset and 8192 for the institute-provided dataset
Backbone depth and width	18 self-weighted blocks, 200 output channels per block	The first block projects 2 input channels to 200 channels; later blocks keep the same width
Branch 1	Conv1D + BN, $C_{l}=200$, k=3, s=1, p=1	Normalized branch output is multiplied by $\lambda_{l}$
Branch 2	Conv1D + BN, $C_{l}=200$, k=1, s=1, p=0	Pointwise branch for channel mixing
Branch 3	Identity/projection + BN, $C_{l}=200$	A 1×1 projection is used only when needed to make the channels compatible
Batch normalization	Running mean/variance enabled, affine parameters disabled	Prevents $\lambda_{l}$ from being absorbed by a branch-wise BN scale parameter
Scaling vector	Channel-wise learnable gain initialized to 1	Excluded from weight decay
Activation and recalibration	ReLU followed by SE block with reduction ratio 16	SE recalibration is applied after branch aggregation
Classifier head	GAP followed by FC layers 200–1000–128–12	Dropout p=0.15 after the first two FC layers

**Table 6:** Training and model-selection configuration.

Item	Setting
Loss function	Categorical cross-entropy on logits
Optimizer	Adam, $\beta_{1}=0.9$, $\beta_{2}=0.999$, $\epsilon =10^{-8}$
Initial learning rate	$1\times 10^{-3}$ for training from scratch and baseline models; $1\times 10^{-4}$ for fine-tuning
Learning-rate schedule	Cosine annealing, $T_{\max}=30$ epochs, $\eta_{\min}=1\times 10^{-6}$
Batch size	128
Maximum epochs	30
Weight decay	$1\times 10^{-4}$ for convolutional and fully connected weights; zero for bias and $\lambda_{l}$ parameters
Batch-normalization setting	Running mean/variance enabled; affine scale and shift disabled in self-weighted branches
Checkpoint selection	Model with the lowest validation loss retained for final testing
Convergence epoch	Epoch at which the best validation loss was first reached
Baseline protocol	Same data splits, optimizer, schedule, batch size, maximum epochs, and checkpoint rule as the proposed model
Transfer protocol	Source model initialized from the broader-parameter setting; all trainable layers fine-tuned on the target setting

**Table 7:** Accuracy (%) for different backbone and fully connected depths.

	FC Layers	1	2	3
Backbone Blocks				
1		41.47	76.19	76.40
2		62.36	83.43	84.75
3		64.57	89.93	89.65
4		53.02	89.68	**91.17**
5		59.60	90.82	90.98

**Table 8:** Feature-head configuration.

Layer	Parameters	Input Shape	Output Shape
Conv1D	2→200, k=50, s=50	(B,2,10,000)	(B,200,200)
GAP	–	(B,200,200)	(B,200,1)
Flatten	–	(B,200,1)	(B,200)
Linear	200→1000	(B,200)	(B,1000)
ReLU, dropout	p=0.15	(B,1000)	(B,1000)
Linear	1000→128	(B,1000)	(B,128)
ReLU, dropout	p=0.15	(B,128)	(B,128)
Linear	128→12	(B,128)	(B,12)

**Table 9:** Performance for different backbone depths.

Blocks	Accuracy (%)	Average Validation Loss
1	76.40	0.5357
2	84.75	0.3525
3	89.65	0.2439
4	91.17	0.2187
5	90.98	0.2184
6	90.95	0.2304
7	91.03	0.2100
8	91.69	0.2224
9	91.54	0.2008
10	91.40	0.2158
12	91.86	0.2106
18	**91.90**	**0.1936**

**Table 10:** Training-from-scratch and transfer results on the 100 kHz dataset.

Strategy	Best Validation Loss	Convergence Epochs	Test Accuracy (%)
Training from scratch on target dataset	**0.1936**	21	**91.91**
Fine-tuning on small target-domain subset	0.1948	**5**	91.81

**Table 11:** Performance of the evaluated architectures on the synthetic dataset.

Metric	Proposed	Transfer	Inception	CaiT	ViT for Small Data	ResNet50
Best validation loss	**0.1936**	0.1948	0.2094	0.3779	0.5146	0.7334
Average accuracy (%)	**91.91**	91.81	91.39	84.02	78.86	75.75

**Table 12:** Performance on the institute-provided IQ dataset.

Method	Average Test Accuracy (%)
Proposed network	**93.61**
Transfer network	92.75
Inception	92.32
CaiT	87.96
ViT for small data	83.55
ResNet50	78.14

## Data Availability

The minimum validation dataset for the MATLAB 2021-generated synthetic IQ data has been uploaded as supporting material. Restrictions apply to the availability of the institute-provided IQ dataset. These data were obtained from a third party and are not publicly available because of privacy reasons.
